# Comparative Evaluation of Prevalence of Upper Cervical Vertebrae Anomalies in Cleft Lip/Palate Patients: A Retrospective Study

**DOI:** 10.5005/jp-journals-10005-1258

**Published:** 2015-02-09

**Authors:** Sanjeev Datana, Ashish Bhalla, Prasanna Kumar, Supriya Kumar Roy, Sanjay Londhe

**Affiliations:** Classified Specialist, Department of Orthodontics and Dentofacial Orthopedics Army Dental Centre (R & R), New Delhi, India; Graded Specialist, Department of Pedodontics, Army Dental Centre (R & R) New Delhi, India; Classified Specialist, Department of Orthodontics and Dentofacial Orthopedics Armed Forces Medical College, Pune, Maharashtra, India; Classified Specialist, Department of Oral and Maxillofacial Surgery, Army Dental Centre (R & R), New Delhi, India; Super Specialist and Consultant, Department of Orthodontics and Dentofacial Orthopedics Army Dental Centre (R & R), New Delhi, India

**Keywords:** Cleft lip and palate, Upper cervical vertebrae anomalies, Lateral cephalogram.

## Abstract

**Purpose:** The patients with cleft lip and palate have a higher risk of cervical vertebrae anomalies than do patients in general population. The aim of present study was to determine the prevalence of various upper cervical spine anomalies in different type of clefts.

**Procedures:** Lateral cephalograms of 128 patients (66 males, 62 females) with cleft lip and palate, and 125 (60 males, 65 females) non syndromic patients without cleft lip and palate were selected at random from archive. Cephalograms of the patients were traced and the diagnosis of any cervical vertebrae anomaly was noted. Anomalies were categorized as either: posterior arch deficiency or fusions.

**Main findings:** Prevalence of cervical vertebrae anomalies in the c lef t group was 20. 3% while it was 6.4% in the control group. Further cervical vertebrae anomalies were 16.6% in the CPO group, 19.1% in the BCLP group, and 22.2% in the UCLP group.

**Conclusion:** A higher prevalence of cervical vertebrae anomalies was observed in cleft lip and palate patients. The prevalenc e obser ved is 3 times more in clef t group than c ontrol group.

**How to cite this article:** Datana S, Bhalla A, Kumar P, Roy SK, Londhe S. Comparative Evaluation of Prevalence of Upper Cervical Vertebrae Anomalies in Cleft Lip/Palate Patients: A Retrospective Study. Int J Clin Pediatr Dent 2014;7(3):168-171.

## INTRODUCTION

Development of the head and face comprises one of the most complex events during embryonic development, coordinated by a network of transcription factors and signaling molecules together with proteins conferring cell polarity and cell-cell interactions. Disturbance of this tightly controlled cascade can result in a facial cleft where the facial primordia ultimately fail to meet and fuse or form the appropriate structures. Collectively, craniofacial abnormalities are among the most common features of all birth defects. The most frequent of these are the orofacial clefts, cleft lip and/or cleft palate (CLP). CLP results in complications affecting feeding, speech, hearing and psychological development.^1,2^

The patients with cervical spine anomalies have a higher risk of developing CLP than do patients in general population.^3^ The radiographic appearance of anomalies of cervical vertebrae has been described by several authors.^4,5^ The aim of present study was to determine the prevalence of various upper cervical spine anomalies in different type of clefts.

## MATERIALS AND METHODS

**Fig. 1:**
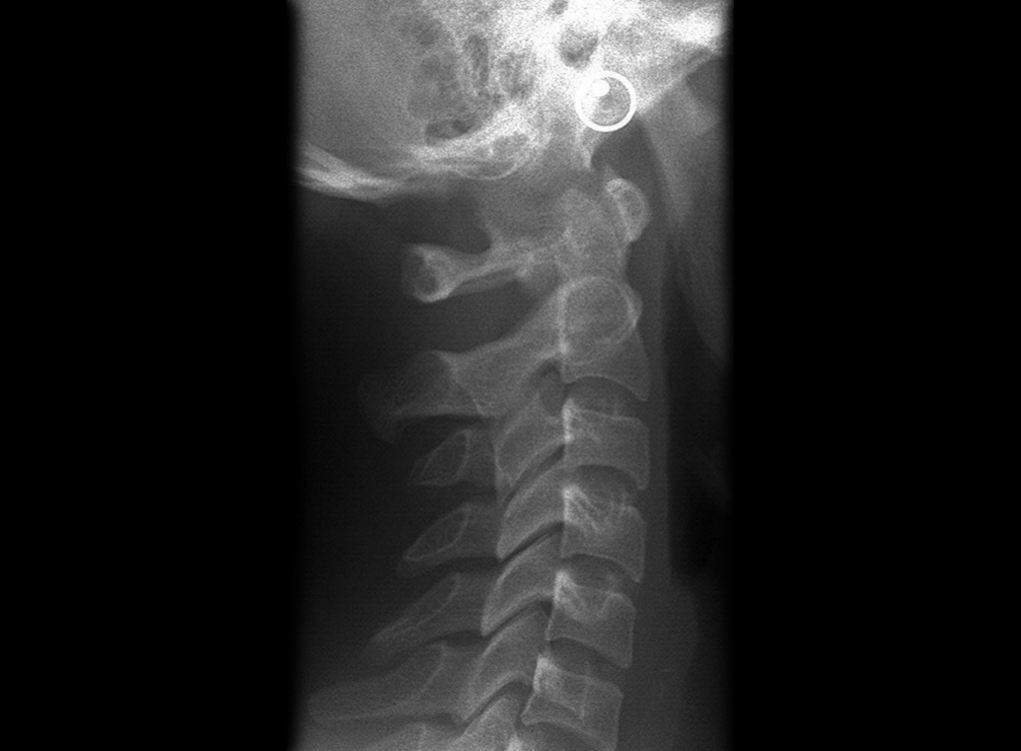
Normal entire cervical spine

Lateral cephalometric radiographs of 128 patients (66 males, 62 females) with cleft, aged 6 years or older were chosen from archive of records of patient being treated in ortho department of tertiary care dental centre. Records of Lateral cephalographs showing the entire cervical spine were selected (Fig. 1). Categorization of CLP types was based on primary and secondary palatal schemes, with cleft type inclusion as follows: cleft palate only (CPO), bilateral cleft lip and palate (BCLP) and unilateral cleft lip and palate (UCLP). The sample with cleft consisted of 3 groups: 18 patients with CPO (7 males, 11 females), 47 patients with BCLP (21 males, 26 females), and 63 patients with UCLP (38 males, 25 females). The lower age limit of 6 years was selected because malformations or anomalies of the upper cervical vertebrae cannot be confirmed at an earlier age 6. The control group (nonsyndromic patients without cleft) for the present study consisted of 125 patients (60 males, 65 females) selected at random from archive of records of patient being treated in ortho department. The study material is presented in Tables 1 and 2.

Lateral radiographs of the subjects were traced on acetate paper, and following the careful examination of the radiographs and tracings, the diagnosis of any cervical vertebrae anomalies were noted. Anomalies were categorized as either: (1) posterior arch deficiency (PAD) categorized into spina bifda or dehiscence or (2) fusions (FUS) categorized into fusion between two vertebras, block fusion, or occipitalization of the atlas.

## RESULTS

**Graph 1:**
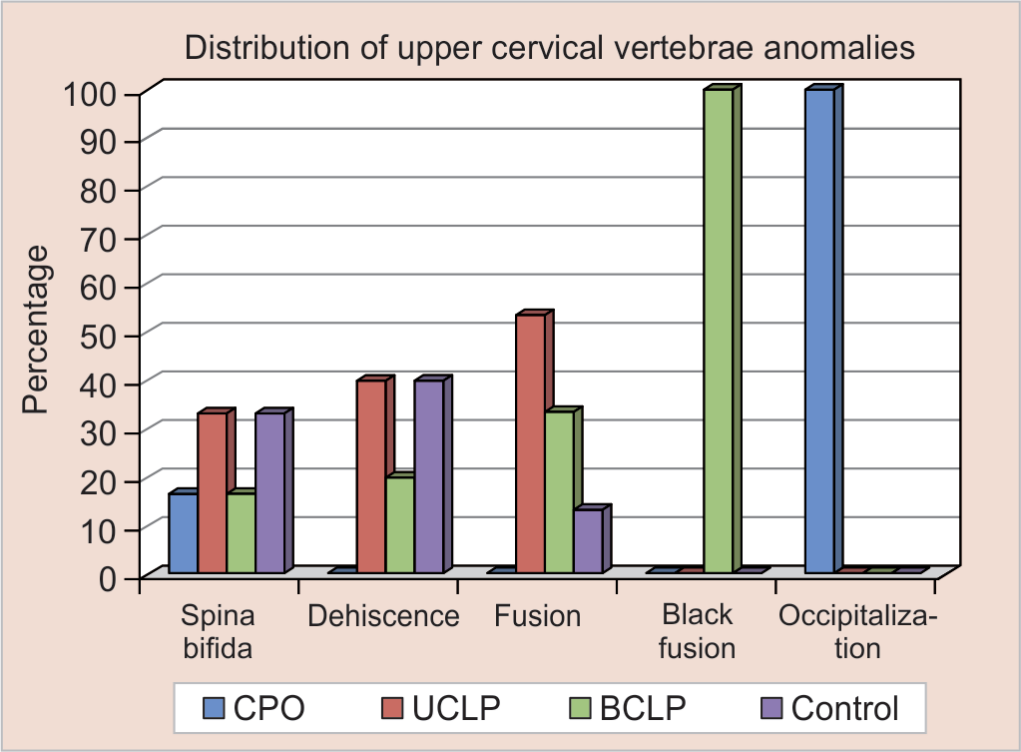
The different kinds of cervical vertebrae anomalies in each of the four study groups

In the cleft group, thrice as many individuals (20.3%) had cervical vertebrae anomalies when compared with the control group (6.4%) (Table 3). In the group with cleft, fusions were more common (11.7%) than posterior arch deficiencies (8.5%) while, and in the control group PAD were more common (4.8%). The occurrence of the cervical vertebrae anomalies was 16.6% in the CPO group, 19.1% in the BCLP group, and 22.2% in the UCLP group. The different kinds of cervical vertebrae anomalies in each of the four study groups are listed in Table 4 (Graph 1). Fusion was most prevalent in UCLP group while spina bifda and dehiscence were equally distributed in UCLP and control group. Occipitalization was seen only in CPO group and block fusion in BCLP group.

**Table 1:** **Table 1:** Study sample

		*Male*				*Female*				*Total*	
		*N*		*%*		*N*		*%*		*N*	
Cleft group		66		(51.5)		62		(48.4)		128	
Control		60		(48)		65		(52)		125	

**Table 2:** **Table 2:** Cleft sample

		*Male*				*Female*				*Total*			
		*N*		*%*		*N*		*%*		*N*		*%*	
CPO		7		(5.5)		11		(8.5)		18		(14)	
UCLP		38		(29.6)		25		(19.5)		63		(49.2)	
BCLP		21		(16.4)		26		(20.3)		47		(36.7)	

## DISCUSSION

**Fig. 2:**
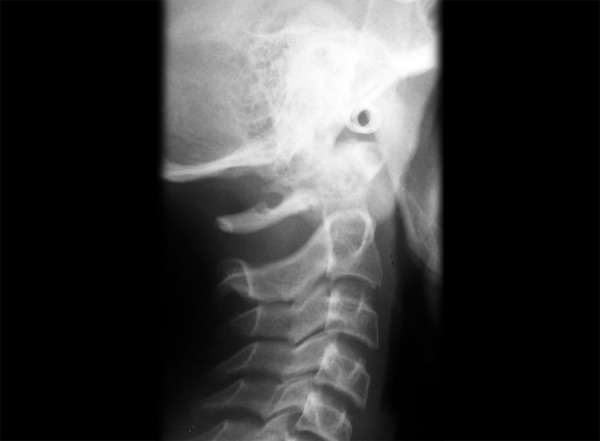
Spina bifda

**Fig. 3:**
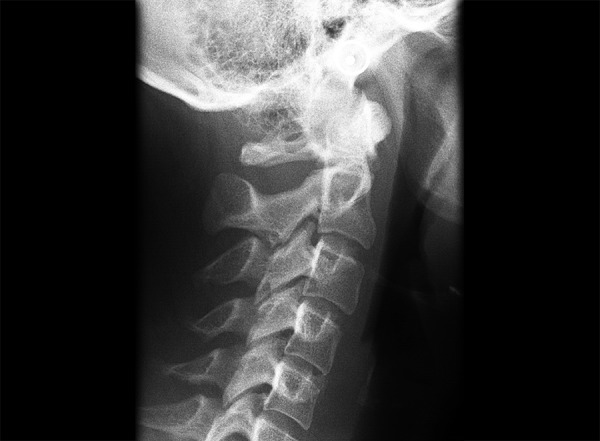
Dehiscence

**Fig. 4:**
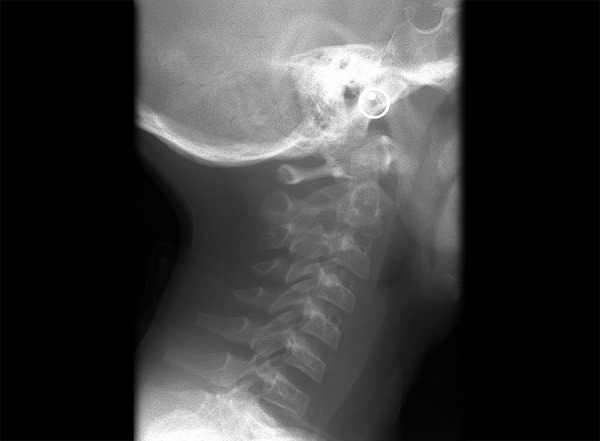
Fusion

**Fig. 5:**
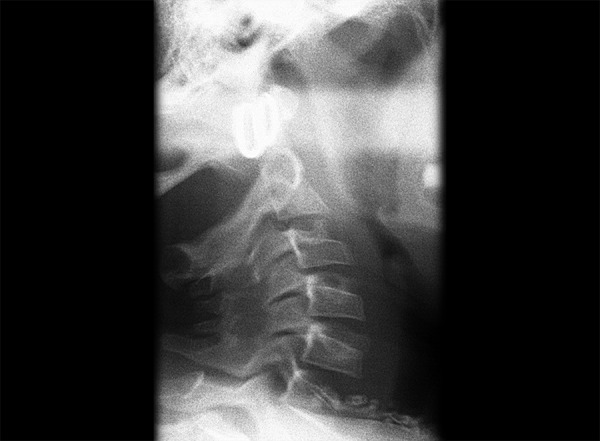
Block fusion

**Fig. 6:**
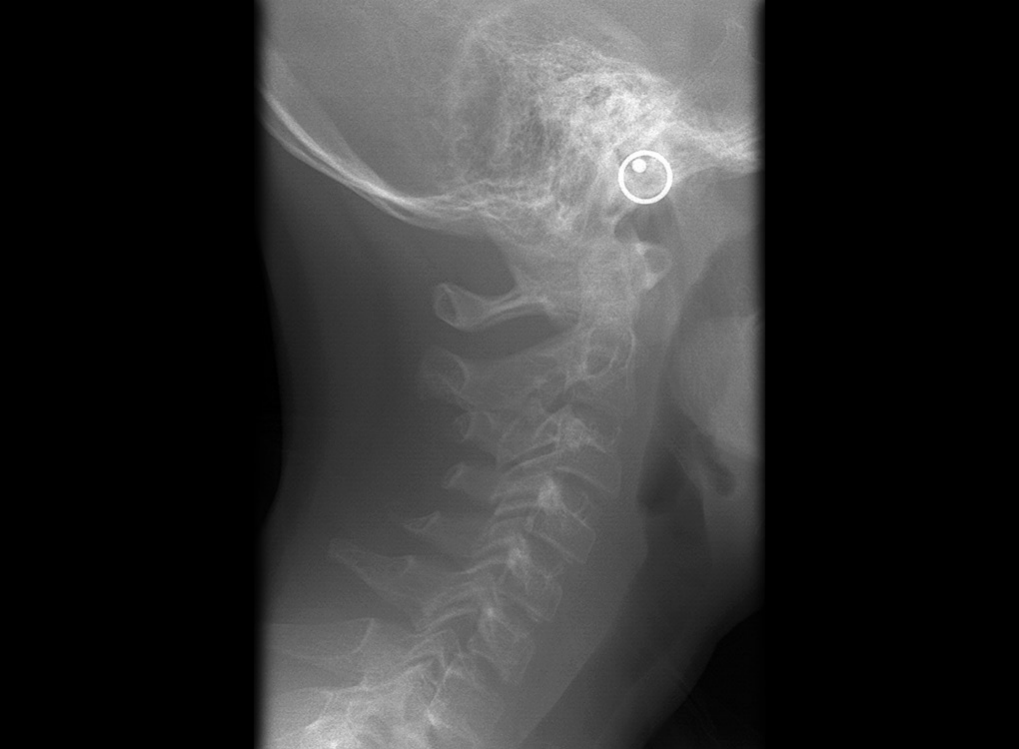
Occipitalization

The cervical vertebrae anomalies are commonly divided into posterior arch deficiencies (PAD) and fusions (FUS).^6-8^ Posterior arch deficiencies are subdivided into spina bifda, (Fig. 2) which implies incomplete ossification in the spinous process and generally occurs in the posterior arch of the vertebral unit, and dehiscence, (Fig. 3) which implies incomplete development of the structures. Dehiscence in the atlas affects either the anterior arch or the posterior arch, posterior arch dehiscence being most common in the midline. Fusion (Fig. 4) is bony union of one unit with another at the articulation facets, neural arch, or transverse processes and may be subdivided into fusion between two cervical vertebrae; block fusion (Fig. 5) in which the bony union includes the vertebral bodies; and occipitalization, (Fig. 6) the assimilation of the atlas to the base of the skull or atlantooccipital fusion or some degree of bony union between the skull and the atlas.

The upper cervical anomalies in the present sample were more common in cleft group than control. The prevelance of anomalies in the present sample was 20.6% similar to a group of American subjects^9^ with clefts 22% and Norwegian children^10^ with cleft lip, cleft palate, or both 18.2%, but higher than that found in Scottish children,^6^ who had a prevalence of 13.6%.

**Table 3:** **Table 3:** Cervical vertebrae anomalies in cleft and control sample

		*N*		*PAD*				*Fusion*				*Total number*			
				*N*		*%*		*N*		*%*		*N*		*%*	
CPO		18		2		(11.1)		1		(5.5)		3		(16.6)	
UCLP		63		6		(9.5)		8		(12.6)		14		(22.2)	
BCLP		47		3		(6.3)		6		(12.7)		9		(19.1)	
Total cleft group		128		11		(8.5)		15		(11.7)		26		(20.3)	
Control group		125		6		(4.8)		2		(1.6)		8		(6.4)	

**Table 4:** **Table 4:** Different type of cervical vertebrae anomalies

		*Spina bifda*				*Dehiscence*				*Fusion*				*Block fusion*				*Occipitalization*			
		*N*		*%*		*N*		*%*		*N*		*%*		*N*		*%*		*N*		*%*	
CPO		2		(16.6)		–		–		–				–				1		(100)	
UCLP		4		(33.3)		2		(40)		8		(53.3)		–				–			
BCLP		2		(16.6)		1		(20)		5		(33.3)		1		(100)		–			
Control		4		(33.3)		2		(40)		2		(13.3)		–				–			

In the present sample with cleft, fusions were more common than posterior arch deficiencies when compared to control which was in lines with study by Ugar and Semb^10^ while posterior arch deficiencies were more common in cleft sample of Sandham.^6^

The prevalence of upper cervical anomalies in cleft patients indicates a significant association between the two and this could improve the screening of these patients at all levels as treatment of cleft lip and palate requiremulti disciplinary approach. Also, the significant prevalence of these anomalies suggests certain common embryonic pathway for these malformations to co-exist.

## CONCLUSION

Within limitation of present study conclusion made are:

1. The results of this study confirm an association between cleft lip and palate and cervical vertebrae anomalies.
2. Cervical vertebrae anomalies occurred 3 times more frequently in subjects with clefts than control group.
3. The prevalence of anomalies was found to be significantly greater in the UCLP sample.
4. Fusion appear to be more closely associated with UCLP and BCLP while occipitalization was found only in CPO.

Further studies are required to establish association between oral cleft and upper cervical anomalies at the genetic level.
